# HALP Score in Predicting Post-Liver Transplant Outcomes in Patients with Hepatocellular Carcinoma

**DOI:** 10.3390/jcm15083011

**Published:** 2026-04-15

**Authors:** Sertac Usta, Fuat Aksoy, Yasin Dalda, Volkan Ince, Harika G. Bag, Brian I. Carr, Sezai Yilmaz

**Affiliations:** 1 Department of Surgery, Liver Transplantation Institute, Inonu University, Malatya 44280, Türkiye; yasin.dalda@inonu.edu.tr (Y.D.); volkan.ince@inonu.edu.tr (V.I.); brianicarr@hotmail.com (B.I.C.); sezai.yilmaz@inonu.edu.tr (S.Y.); 2 Department of Surgery, Faculty of Medicine, Uludag University, Bursa 16059, Türkiye; fuataksoy@uludag.edu.tr; 3 Department of Biostatistics, Faculty of Medicine, Inonu University, Malatya 44280, Türkiye; harika.gozukara@inonu.edu.tr

**Keywords:** HALP, HCC, LDLT, albumin, relapse, hepatoma, hepatic cancer, liver tumor

## Abstract

**Background:** Accurate prognostic stratification remains essential for optimizing outcomes in hepatocellular carcinoma (HCC) patients undergoing liver transplantation (LT). The hemoglobin–albumin–lymphocyte–platelet (HALP) score is a composite biomarker reflecting systemic inflammation, nutritional status, and immune competence, and has demonstrated prognostic value in several malignancies. This study aimed to evaluate the predictive utility of the HALP score for survivals and recurrence in HCC patients undergoing LT. **Methods:** A total of 476 consecutive patients who underwent LT for HCC between 2006 and 2024 were retrospectively analyzed. Pretransplant HALP scores were calculated for all patients. Receiver operating characteristic (ROC) analysis identified an optimal cut-off value of 29 for recurrence prediction. Patients were stratified into HALP ≥ 29 and HALP < 29 groups. DFS and recurrence rates were compared. Prognostic performance was assessed using the concordance index (C-index) and area under the ROC curve (AUC). Outcomes were further compared with the Milan and Expanded Malatya criteria. **Results:** Of the 476 patients, 335 (70.4%) had HALP ≥ 29 and 141 (29.6%) had HALP < 29. The HALP ≥ 29 group demonstrated significantly higher 5- and 10-year DFS rates compared with the HALP < 29 group (67.1% vs. 58.5% and 49.5% vs. 33.5%, respectively; *p* < 0.001). Recurrence rates were significantly lower in the HALP ≥ 29 group (14.0% vs. 31.9%; *p* < 0.001). However, patients within the Milan and Expanded Malatya criteria showed superior long-term DFS and lower recurrence rates in the HALP ≥ 29 compared to the HALP < 29 group (*p* ≤ 0.037). HALP ≥ 29 was associated with lower tumor burden parameters and improved hepatic functional reserve. Despite its significance, HALP demonstrated inferior discriminative performance (C-index: 0.565) compared with the Milan (0.621) and Expanded Malatya (0.648) criteria. Patients beyond the Milan criteria (*n* = 233) with HALP ≥ 29 achieved a 5-year overall survival of 54.2%, compared with 37.8% with HALP < 29. **Conclusions:** Low HALP score is associated with poor DFS and a high post-transplant recurrence rate. Although it represents a non-invasive and cost-effective biomarker, its prognostic accuracy remains inferior to established transplant selection criteria, limiting its use as a standalone selection tool. However, individuals beyond Milan with HALP ≥ 29 achieved survival outcomes exceeding internationally accepted post-transplant benchmarks. Incorporating HALP into pre-transplant evaluation may help identify a biologically favorable subgroup among patients traditionally considered high risk based solely on tumor burden.

## 1. Introduction

Hepatocellular carcinoma (HCC) is the sixth most common cancer, and the third leading cause of cancer-related death remains a global health concern. It is estimated that there will be 1.41 million new cases and 1.26 million HCC-associated deaths in 2045 [1]. Eligibility for LT in HCC patients is often guided by established radiological criteria such as the Milan or UCSF criteria, which aim to select candidates with limited tumor burden and favorable biology to minimize recurrence risk and maximize post-transplant survival [2,3]. However, recurrence of HCC after LT remains a significant concern, occurring in approximately 10–20% of patients, and is associated with poor prognosis [4]. While tumor size, number, and vascular invasion are commonly used predictors, they do not fully capture the biological heterogeneity of HCC or the systemic condition of the host. There is, therefore, a growing need to identify reliable and easily accessible biomarkers that reflect both tumor aggressiveness and host-related factors, such as systemic inflammation, immune competence, and nutritional status [5,6,7,8].

In recent years, composite prognostic indices derived from routine laboratory tests have gained attention for their ability to integrate multiple dimensions of host physiology. One such index is the Hemoglobin, Albumin, Lymphocyte, and Platelet (HALP) score. This biomarker, calculated as hemoglobin (g/L) × albumin (g/L) × lymphocyte count (/L) ÷ platelet count (/L), provides a simple yet comprehensive reflection of the patient’s immune–nutritional and inflammatory state [9]. Each component of the HALP score has been individually associated with cancer outcomes: anemia and hypoalbuminemia reflect poor nutritional and functional status; lymphopenia indicates impaired cell-mediated immunity; and thrombocytosis may signify tumor-associated inflammation or pro-metastatic activity [10,11,12]. The HALP score consolidates these variables into a single, objective, and reproducible measure.

The prognostic value of the HALP score has been demonstrated across various malignancies, particularly within the gastrointestinal cancer spectrum. Multiple studies have shown that a lower HALP score is associated with poorer overall survival (OS), increased recurrence risk, and worse pathological features in gastric, colorectal, pancreatic, and esophageal cancers [13,14,15,16]. Despite this growing evidence, studies on the application of the HALP score in the field of transplantation oncology, particularly in HCC patients undergoing liver transplantation, are not satisfactory [17,18]. This represents a critical gap in the literature, especially considering that the systemic status of liver transplant candidates can significantly affect perioperative risk, immunosuppression tolerance, and long-term oncological outcomes.

Moreover, identifying non-invasive biomarkers that can be easily integrated into pre-transplant evaluation may help refine candidate selection, risk stratification and surveillance protocols in clinical practice. The HALP score, given its simplicity and low cost, holds potential to serve as such a tool. However, its clinical utility in the context of LT has yet to be established.

This study aims to address this unmet need by evaluating the prognostic significance of the HALP score in predicting OS and disease-free survival (DFS) in a well-defined cohort of HCC patients undergoing liver transplantation. By focusing on this unique and clinically significant population, our study provides evidence for the utility of the HALP score in the transplantation oncology setting by comparing it to previously established transplant criteria, such as the Milan [2], Malatya [7] and Expanded Malatya criteria [8,19]. We aimed to investigate whether the HALP score, as an indicator of systemic inflammatory and nutritional status, could provide additional prognostic information beyond traditional tumor-based criteria and whether it could be integrated into comprehensive pre-transplantation assessment frameworks.

## 2. Materials and Methods

### 2.1. Study Design and Patient Population

This retrospective cohort study included 476 patients diagnosed with HCC who underwent liver transplantation, consecutively at Inonu University, Liver Transplantation Institute between March 2006 and December 2023. Inclusion criteria were patients with post-transplant follow-up period longer than 90 days, and HCC diagnosis confirmed by explant histopathology. Patients with combined HCC and cholangiocarcinoma, and missing laboratory data required for HALP score calculation were excluded.

### 2.2. Data Collection and HALP Score Calculation

Preoperative laboratory tests performed within 30 days prior to transplantation were retrieved from the institution’s prospectively and consecutively recorded database. The HALP score was calculated using the formula: hemoglobin (g/L) × albumin (g/L) × lymphocyte count (/L) ÷ platelet count (/L), as described in prior studies [10]. Demographic, clinical, and pathological data were also collected.

### 2.3. Statistical Analysis

Statistical analyses were performed using IBM SPSS Statistics version 28.0 (IBM Corp., Armonk, NY, USA). The normality of the quantitative data was assessed by the Kolmogorov–Smirnov test. Two independent group comparisons were performed by the Mann–Whitney U test. Median, minimum, and maximum values were used to summarize the quantitative data. Distributions of the qualitative data were presented by count and percentage. Qualitative variables were compared using the Pearson’s chi-square test or continuity-corrected chi-square test. Receiver Operating Characteristic (ROC) curve analysis was performed in SPSS to evaluate the predictive performance of the HALP score for OS and DFS, calculating the area under the curve (AUC) with 95% confidence intervals (CI). Optimal cut-off values were determined by maximizing the Youden index to balance sensitivity and specificity. A two-tailed *p*-value < 0.05 was considered statistically significant.

The Kaplan–Meier method was used for survival estimations, and the Log-Rank test was used for survival comparisons between groups. Univariable and multivariable (with forward selection method) Cox regression analysis was used to obtain the Hazard Ratios. The prognostic abilities were given by using concordance index (C-index) and area under the ROC curve (AUC). The C-index analysis was conducted using Jamovi (version 2.7.6). In all analyses, the two-sided significance level was considered as <0.05.

### 2.4. Ethics Statement

The study protocol was approved by the Inonu University Scientific Research and Publication Ethics Board (Approval Number: 2024/6547).

## 3. Results

### 3.1. Patient Population

A total of 476 liver transplant recipients with hepatocellular carcinoma (HCC) were included in the study. The median age of the patients was 56 years (1–72), 412 were male (86.6%), the most frequent etiology was viral hepatitis *(n* = 362, 76.1%), 394 had AFP ≤ 200 ng/mL (83.7%), 243 were within the Milan criteria (51.1%), 325 were within the Expanded Malatya criteria (68.3%). Post-transplant recurrence rate was 19.3% (*n* = 92). Baseline demographic, clinical, and pathological features of the cohort are summarized in Table A1.

### 3.2. Determination of HALP Patient Groups

HALP scores were calculated. Receiver operating characteristic (ROC) analysis was performed to determine the optimal HALP cut-off value for predicting recurrence. The optimal threshold identified by the Youden index was ≤28.9 (J = 0.25). At this dataset-specific cut-off value, sensitivity was 50.0% and specificity was 75.0%. Patients were then categorized into two groups based on pre-transplant HALP score: <29 (*n* = 141) and ≥29 (*n* = 335) (Table A2).

### 3.3. Survival Analysis and Recurrence

#### 3.3.1. Comparison of Overall Survival and Disease-Free Survival Between Groups

**Figure 1:**
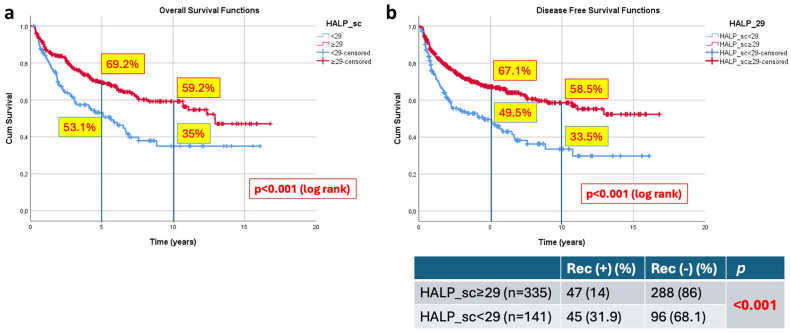
Survivals of all cohorts according to HALP groups (*n* = 476). (**a**) Overall survivals; (**b**) Disease-free survivals and recurrence rates.

Kaplan–Meier survival analysis demonstrated that patients with HALP ≥ 29 had significantly superior long-term outcomes compared with those with HALP < 29. The 5- and 10-year OS rates were 69.2% and 59.2% in the HALP ≥ 29 group, compared with 53.1% and 35.0% in the HALP < 29 group, respectively (Figure 1a).

Similarly, the 5- and 10-year DFS rates were 67.1% and 58.5% in the HALP ≥ 29 group, whereas they were 49.5% and 33.5% in the HALP < 29 group, respectively. The differences between the groups were statistically significant (log-rank *p* < 0.001) (Figure 1b).

On univariate Cox regression analysis, HALP < 29 was associated with poorer OS (HR: 1.79, 95% CI: 1.32–2.42, *p* < 0.001) and DFS (HR: 2.7, 95% CI: 1.78–4.1, *p* < 0.001) (Table 1).

In multivariable Cox regression, when all variables were included in the model without using the variable selection method, the HALP score was not statistically significant (OS; HR: 1.076, 95% CI: 0.669–1.730, *p* = 0.762), DFS; (HR: 1.066, 95% CI: 0.659–1.727, *p* = 0.794). Expanded Malatya criteria, recurrence and ALT level were significant predictors of DFS, and Expanded Malatya criteria, recurrence status and NLR were the significant predictors of OS (Table 2).

#### 3.3.2. Recurrence Analysis

During follow-up, 92 of 476 patients (19.3%) experienced post-transplant HCC recurrence. Patients with HALP < 29 had a significantly higher recurrence rate than those with HALP ≥ 29 (45/141, 31.9% vs. 47/335, 14.0%, respectively; *p* < 0.001), corresponding to a more than two-fold absolute difference in recurrence incidence (Figure 1b).

When time-to-recurrence was analyzed, HALP < 29 was associated with an increased risk of recurrence (HR: 2.7, 95% CI: 1.79–4.1, *p* < 0.001).

### 3.4. Comparison of Demographic, Clinical, and Pathological Characteristics Between Groups

To better understand the underlying factors contributing to the prognostic performance of the HALP score for survival and recurrence, patients were compared according to HALP stratification.

Patients with HALP ≥ 29 demonstrated significantly more favorable tumor and inflammatory profiles compared with those with HALP < 29. The median largest tumor diameter was significantly lower in the HALP ≥ 29 group (2.8 cm vs. 4.0 cm). Inflammatory indices were also significantly reduced in the high-HALP group, including the neutrophil-to-lymphocyte ratio (NLR) (2.2 vs. 4.31) and the platelet-to-lymphocyte ratio (PLR) (65.4 vs. 160.82).

In terms of liver function, a significantly higher proportion of patients with HALP ≥ 29 had MELD-Na < 15 (75% vs. 25%) and were classified as Child–Pugh class A (82.4% vs. 17.6%). Moreover, patients with higher HALP scores were more frequently within established transplant selection criteria. The proportion of patients meeting the Milan criteria was significantly higher in the HALP ≥ 29 group (76.1% vs. 29.9%). Similarly, eligibility within the Malatya criteria (77.1% vs. 22.9%) and the Expanded Malatya criteria (76.0% vs. 24.0%) was significantly greater among patients with HALP ≥ 29 (all *p* < 0.05) (Table 3).

### 3.5. Prognostic Performance of HALP ≥ 29

We demonstrated that the HALP score is a statistically significant parameter for predicting post-transplant prognosis in patients with HCC. However, to better contextualize its clinical utility, the prognostic performance of HALP ≥ 29 was compared with established transplant selection criteria.

#### 3.5.1. HALP ≥ 29 vs. Within Milan Criteria

**Figure 2:**
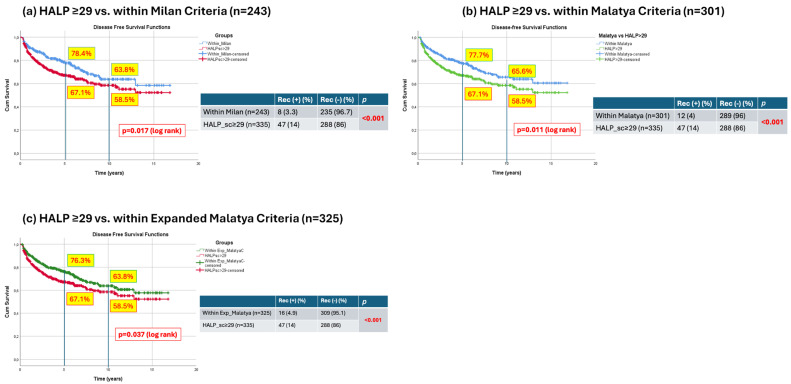
Comparison of DFS and recurrence rates between HALP > 29 (*n* = 335) and within the Tx Criteria. (**a**) HALP ≥ 29 vs. within Milan Criteria (*n* = 243); (**b**) HALP ≥ 29 vs. within Malatya Criteria (*n* = 301); (**c**) HALP ≥ 29 vs. within Expanded Malatya Criteria (*n* = 325).

Patients meeting the Milan criteria had superior long-term DFS compared with those classified as HALP ≥ 29. The 5- and 10-year DFS rates were 78.4% and 63.8% in patients within the Milan criteria, whereas they were 67.1% and 58.5% in patients with HALP ≥ 29 (log-rank *p* = 0.017). Recurrence rates were significantly lower among patients within the Milan criteria compared with those with HALP ≥ 29 (3.3% vs. 14.0%, respectively; *p* < 0.001) (Figure 2a).

#### 3.5.2. HALP ≥ 29 vs. Within Malatya Criteria

Similarly, patients meeting the Malatya criteria demonstrated superior DFS compared with the HALP ≥ 29 group. The 5- and 10-year DFS rates were 77.7% and 65.6% in the Malatya group, versus 67.1% and 58.5% in the HALP ≥ 29 group (log-rank *p* = 0.011) (Figure 2b). Recurrence rates were also significantly lower in patients within the Malatya criteria compared with the HALP ≥ 29 group (4% vs. 14.0%, respectively; *p* < 0.001) (Figure 2b).

#### 3.5.3. HALP ≥ 29 vs. Within Expanded Malatya Criteria

Similarly, patients meeting the Expanded Malatya criteria demonstrated superior DFS compared with the HALP ≥ 29 group. The 5- and 10-year DFS rates were 76.3% and 63.8% in the Expanded Malatya group, versus 67.1% and 58.5% in the HALP ≥ 29 group (log-rank *p* = 0.037) (Figure 2c). Recurrence rates were also significantly lower in patients within the Expanded Malatya criteria compared with the HALP ≥ 29 group (4.9% vs. 14.0%, respectively; *p* < 0.001) (Figure 2c).

### 3.6. Discriminative Performance

**Figure 3:**
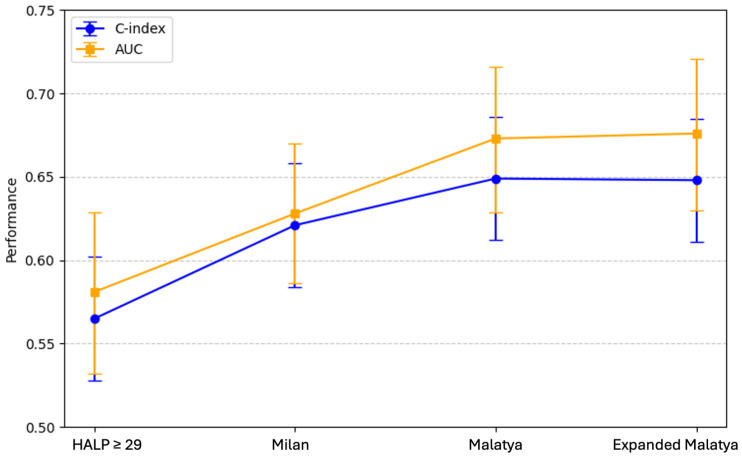
Comparison graphic of discriminative performance of selection criteria for HCC.

Prognostic discrimination was evaluated using the concordance index (C-index) and area under the receiver operating characteristic curve (AUC). Although HALP ≥ 29 was significantly associated with longer DFS, its discriminative performance was inferior to established transplant criteria. The C-index was 0.565 for HALP, compared with 0.621 for the Milan criteria, 0.649 for the Malatya and 0.648 for the Expanded Malatya criteria (Table A3). On the ROC comparison, the Expanded Malatya criteria achieved the highest predictive performance, as indicated by the AUC (Figure 3).

### 3.7. Can HALP > 29 Be Used as a Patient Selection Criteria for LT in HCC Patients?

#### 3.7.1. HALP Score in Patients Beyond the Transplant Criteria

**Figure A1:**
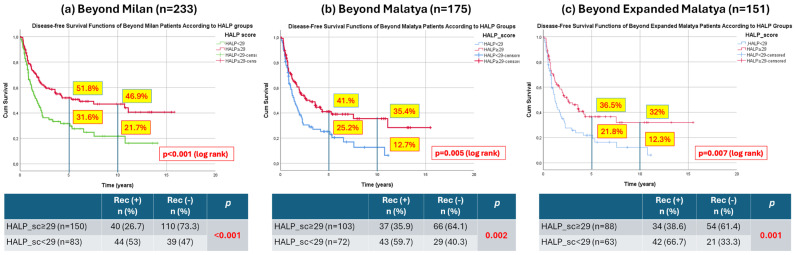
Comparison of DFS and recurrence rates of the HALP groups in patients Beyond the Tx Criteria. (**a**) HALP ≥ 29 vs. HALP < 29 in patients Beyond Milan (*n* = 233), (**b**) HALP ≥ 29 vs. HALP < 29 in patients Beyond Malatya (*n* = 175), (**c**) HALP ≥ 29 vs. HALP < 29 in patients Beyond Expanded Malatya Criteria (*n* = 151).

We next evaluated whether the HALP score could further stratify outcomes among patients who did not meet established transplant criteria. Patients beyond each set of criteria were divided into two subgroups according to their HALP score: high (HALP ≥ 29) and low (HALP < 29). Overall survival (OS), disease-free survival (DFS), and recurrence rates were compared between these subgroups. DFS and recurrence analyses are presented in Figure A1.

#### 3.7.2. Overall Survival Comparison of HALP Subgroups in Beyond Milan Patients

**Figure 4:**
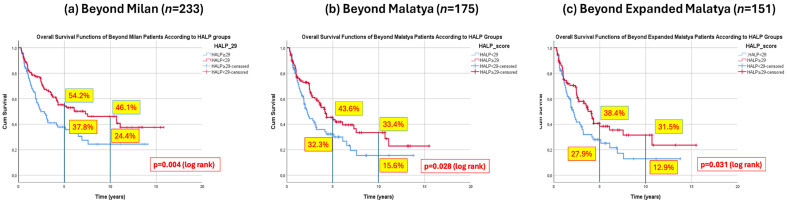
Comparison of OS of the HALP groups in patients Beyond the Tx Criteria. (**a**) HALP ≥ 29 vs. HALP < 29 in patients Beyond Milan (*n* = 233); (**b**) HALP ≥ 29 vs. HALP < 29 in patients Beyond Malatya (*n* = 175); (**c**) HALP ≥ 29 vs. HALP < 29 in patients Beyond Expanded Malatya Criteria (*n* = 151).

Among the 233 patients beyond the Milan criteria, OS differed significantly according to the HALP subgroup. Patients with HALP ≥ 29 had 5- and 10-year survival rates of 54.2% and 46.1%, respectively, whereas those with HALP < 29 had corresponding survival rates of 37.8% and 24.4%. This difference was statistically significant (*p* = 0.004) (Figure 4a).

#### 3.7.3. Overall Survival Comparison of HALP Subgroups in Beyond Malatya Patients

In the 175 patients beyond the Malatya criteria, HALP-based stratification also demonstrated a significant difference in OS. The 5- and 10-year survival rates were 43.6% and 33.4% in the HALP ≥ 29 group, compared to 32.3% and 15.6% in the HALP < 29 group (*p* = 0.028) (Figure 4b).

#### 3.7.4. Overall Survival Comparison of HALP Subgroups in Beyond Expanded Malatya Patients

Similarly, among the 151 patients beyond the Expanded Malatya criteria, patients with HALP ≥ 29 showed superior long-term survival. The 5- and 10-year survival rates were 38.4% and 31.5% in the high HALP group, compared with 27.9% and 12.9% in the low HALP group (*p* = 0.031) (Figure 4c).

### 3.8. HALP < 29 in Patients Within Milan Criteria

Given that a high HALP score significantly predicted a survival advantage even among patients beyond established transplant criteria, we further investigated whether the HALP score could identify patients within the Milan criteria who might experience poor post-transplant outcomes. Since a low HALP score was associated with poor prognosis in the overall cohort, we specifically analyzed survival outcomes among patients within the Milan criteria who had HALP < 29.

Among patients within the Milan criteria, 58 had a HALP score < 29. In this subgroup, the 5- and 10-year OS rates were 75.8% and 50.8%, respectively, while the corresponding DFS rates were 75.7% and 50.7%. Notably, only one patient developed recurrence (1/58, 1.7%).

**Figure A2:**
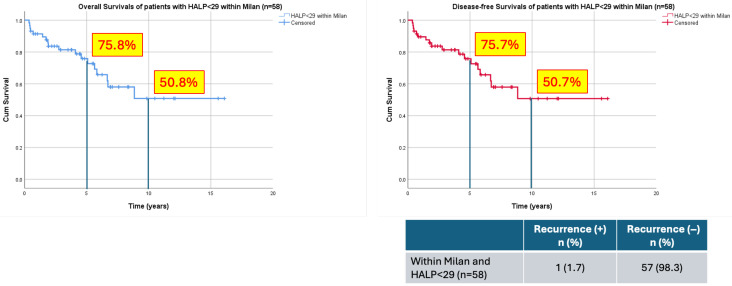
OS, DFS, and recurrence rate of HALP < 29 patients within Milan criteria (*n* = 58).

These findings indicate that a low HALP score did not predict poor outcomes among patients who met the Milan criteria (Figure A2).

## 4. Discussion

This study aimed to evaluate the potential of the HALP score as a prognostic tool for recurrence and DFS in liver transplant recipients with HCC. Our findings demonstrate that higher HALP scores are significantly associated with improved outcomes. Patients with HALP ≥ 29 had significantly longer overall survival (OS: 10.66 ± 0.46 years vs. 7.66 ± 0.67 years; HR 1.789, 95% CI 1.323–2.419; *p* < 0.001) and disease-free survival (DFS: 14.14 ± 0.36 years vs. 10.09 ± 0.74 years; HR 2.708, 95% CI 1.798–4.078; *p* < 0.001) compared to those with HALP < 29 (Table 2). These results suggest that higher HALP scores, indicating better nutritional status and lower systemic inflammation, confer a survival advantage post-transplant. Systemic inflammation and malnutrition are known to promote tumor recurrence and progression, further supporting the clinical relevance of HALP [20,21].

Patients were compared based on HALP stratification to clarify factors affecting its performance for survival and recurrence prediction. Analysis of baseline characteristics (Table 3) revealed that patients with HALP < 29 exhibited larger tumors, higher NLR, PLR, and platelet counts and CHILD and MELD scores, and lower albumin levels (all *p* < 0.05). ALT levels were also elevated, and recurrence was more frequent in this group. We did not identify a significant association between low HALP scores and established adverse prognostic factors, including elevated AFP and GGT levels, vascular invasion, or poor tumor differentiation. However, lower HALP scores were significantly associated with exceeding the Milan, Malatya, and Expanded Malatya criteria, as well as with higher LTD and TTD values, suggesting a relationship between lower HALP and increased tumor burden rather than classical markers of aggressive tumor biology [2,5,7,8,22,23]. Collectively, these findings indicate that higher HALP scores are linked to a less aggressive tumor burden profile (lower LTD and TTD), reduced systemic inflammatory activity (lower NLR and PLR), and better preserved hepatic functional reserve (lower MELD and Child–Pugh scores), underscoring the potential of HALP as an integrative biomarker reflecting both tumor extent and host-related factors.

The higher frequency of viral hepatitis among patients with elevated HALP scores may suggest that individuals with viral hepatitis have a relatively more preserved hematological and nutritional profile compared to other etiologies. This finding could also be explained by the possibility that viral hepatitis patients are diagnosed and followed at earlier stages of disease due to structured surveillance programs, thereby maintaining better laboratory parameters reflected in higher HALP scores. However, this association should be interpreted with caution, particularly if some etiological subgroups include small sample sizes, which may exaggerate apparent differences. Importantly, HALP should not be considered a determinant of etiology but rather a parameter associated with it, and it remains unclear whether this relationship is independent or confounded by other clinical variables.

From a clinical standpoint, the HALP score may be most valuable as an adjunct to existing selection criteria. Its simplicity and accessibility—derived entirely from routine pre-transplant blood tests—make it a practical addition to early risk stratification and post-transplant surveillance planning. Identifying patients with high HALP scores could facilitate intensified follow-up strategies and potential early interventions aimed at mitigating recurrence risk [24]. Multivariable Cox regression analysis (Table 2) identified key independent predictors of OS and DFS. Exceeding the Extended Malatya criteria was associated with worse OS (HR 1.785; 95% CI 1.240–2.571; *p* = 0.002) and DFS (HR 1.907; 95% CI 1.341–2.710; *p* < 0.001), confirming the prognostic value of tumor morphology and burden. HCC recurrence remained the strongest negative predictor for both OS (HR 3.979; 95% CI 2.774–5.708; *p* < 0.001) and DFS (HR 7.342; 95% CI 5.118–10.533; *p* < 0.001). Among laboratory variables, NLR independently predicted OS (HR 1.045; 95% CI 1.011–1.080; *p* = 0.010), and ALT predicted DFS (HR 1.001; 95% CI 1.000–1.001; *p* = 0.021), highlighting the contribution of host inflammatory status and liver function to post-transplant survival [5,7,8,17,18,24]. The HALP score did not reach significance in multivariate analysis. This result can be explained by the fact that the HALP score does not include tumor-related parameters; however, categorizing the HALP score showed a statistically significant relationship with survival. These findings highlight that considering tumor characteristics and host biology together improves prognosis.

Next, the discriminative performance of selection criteria was evaluated (Table A2). While HALP alone had modest predictive ability (C-index 0.565; AUC 0.581), morphological criteria, particularly Malatya and Extended Malatya, demonstrated superior discrimination (C-index 0.648–0.649; AUC 0.673–0.676). On ROC curve analysis, the Expanded Malatya criteria demonstrated the highest predictive performance, as reflected by the largest area under the curve (AUC) (Figure 3), indicating superior discriminative ability compared to the other evaluated criteria. These findings indicate that while HALP reflects clinically meaningful prognostic information, it does not surpass established tumor-based selection models in predicting outcomes after transplantation. This limits its use as an independent decision-making tool. Combining HALP with established criteria or integrating HALP into multiparametric prognostic models may improve patient stratification by incorporating host-related prognostic information.

Subsequently, we performed subgroup analyses to explore whether the HALP score could be used as a patient selection criterion for liver transplantation in patients with more advanced HCC. When survival was analyzed according to HALP score among patients beyond established transplant criteria, those beyond the Milan criteria with HALP ≥ 29 achieved a 5-year overall survival rate of 54.2%, exceeding the widely accepted benchmark of ≥50% 5-year survival considered appropriate for liver transplantation [25,26]. Moreover, in patients beyond the Malatya and Expanded Malatya criteria, HALP ≥ 29 was also associated with a statistically significant survival advantage (Figure 4). These findings suggest that HALP may identify a subgroup of patients beyond conventional morphologic criteria who can still achieve acceptable long-term survival after liver transplantation.

In contrast, among patients within the Milan criteria, those with low HALP scores (<29) demonstrated excellent post-transplant outcomes. In this subgroup of 58 patients, the 5-year overall survival, disease-free survival, and recurrence rates were 75.8%, 75.7%, and 1.7%, respectively (Figure A2). Thus, within the Milan criteria, a low HALP score was not associated with poor prognosis.

Taken together, these results indicate that the prognostic impact of HALP appears to be more pronounced in patients with biologically more aggressive or larger tumor burdens beyond standard criteria. The association between HALP and outcomes is likely confounded by tumor burden and hepatic reserve, as patients with higher HALP had systematically more favorable baseline characteristics.

Limitations of the study are retrospective design, single-center experience, lack of external validation and potential selection bias. The low sensitivity (50%) limits its utility as a screening or selection tool, as a substantial proportion of high-risk patients would not be identified. The HALP score cut-off value is data-driven and may be subject to overfitting, and the discriminative power of the HALP score is low. Additionally, although this dataset is the largest to date examining HALP scores in patients who have undergone liver transplantation due to HCC, external validation of this study’s specific cut-off is required.

## 5. Conclusions

In conclusion, while the HALP score is a valuable indicator of recurrence risk, it does not directly measure tumor burden or biology, and it does not substitute for established selection criteria in predicting long-term post-transplant survival. The Milan and Expanded Malatya criteria remain superior for transplant eligibility assessment. HALP demonstrated significant discriminative value in patients beyond conventional morphologic selection thresholds. Notably, individuals beyond Milan with HALP ≥ 29 achieved survival outcomes exceeding internationally accepted post-transplant benchmarks.

These findings suggest that HALP may serve as a simple, readily available biomarker reflecting systemic inflammatory and nutritional status, thereby complementing tumor morphology in candidate selection. Incorporating HALP into pre-transplant evaluation may help identify a biologically favorable subgroup among patients traditionally considered high risk based solely on tumor burden. HALP can contribute to risk stratification alongside transplant criteria, rather than replacing them.

Prospective validation studies are warranted to determine whether HALP-based stratification can be integrated into future transplant selection algorithms and contribute to more individualized, biology-driven decision-making in liver transplantation for HCC.

## Figures and Tables

**Table 1:** Survival comparison between the HALP groups.

	Overall Survival				Disease-Free Survival			
	Survival (Years) Mean ± SE	Log-Rank *p*-Value	HR (95% CI)	HR *p*-Value	Survival (Years) Mean ± SE	Log-Rank *p*-Value	HR (95% CI)	HR *p*-Value
HALP < 29 (*n* = 141)	7.66 ± 0.67	<0.001	1.789 (1.323–2.419)	<0.001	10.09 ± 0.74	<0.001	2.708 (1.798–4.078)	<0.001
HALP ≥ 29 (*n* = 335)	10.66 ± 0.46		reference		14.14 ± 0.36		reference	

CI: confidence interval; HR: hazard ratio; SE: standard error. HALP ≥ 29 was used as the reference group in Cox regression analysis.

**Table 2:** Multivariable Cox Regression Analysis for Overall Survival and Disease-Free Survival.

		Overall Survival		Disease-Free Survival	
Variable	Category	HR (95% CI)	*p*	HR (95% CI)	*p*
Expanded Malatya Criteria	In	Reference	—	Reference	—
	Out	1.785 (1.240–2.571)	**0.002**	1.907 (1.341–2.710)	**<0.001**
Recurrence	None	Reference	—	Reference	—
	Yes	3.979 (2.774–5.708)	**<0.001**	7.342 (5.118–10.533)	**<0.001**
NLR	—	1.045 (1.011–1.080)	**0.010**	—	—
ALT	—	—	—	1.001 (1.000–1.001)	**0.021**

ALT: alanine aminotransferase; CI: confidence interval; HR: hazard ratio; NLR: neutrophil-to-lymphocyte ratio. Multivariable analysis performed using forward selection Cox proportional hazards model. Bold values indicate statistical significance (*p* < 0.05).

**Table 3:** Comparison of Demographic, Clinical, and Pathological Characteristics Between the HALP Groups.

	HALP < 29			HALP ≥ 29		
	*n*	Median (Min.–Max.)		*n*	Median (Min.–Max.)	*p*
Age at Tx date	141	56 (8–70)		335	56 (1–72)	0.502
BSA, mL/m^2^	141	1.9 (0.84–2.36)		335	1.92 (0.38–2.54)	0.656
BMI, kg/m^2^	141	25.71 (16.1–41)		335	26.1 (14.8–46.88)	0.321
AFP ng/mL	139	11.8 (0.2–14,560)		332	12.45 (0.3–55,000)	0.226
LTD (cm)	141	4 (0–26)		335	2.8 (0.1–18)	0.001
Number of nodules	141	2 (1–20)		335	1 (1–36)	0.194
NLR	141	4.31 (0.99–35.3)		335	2.2 (0.15–8.58)	<0.001
PLR	141	160.82 (78–1092.31)		335	65.4 (2.61–188.82)	<0.001
Platelets, 10^3^/µL	141	115 (23–701)		335	85 (15–373)	<0.001
INR	117	1.31 (0.93–2.6)		265	1.31 (0.82–4.1)	0.488
Creatinine, mg/dL	141	0.78 (0.41–13.8)		335	0.8 (0.37–3.24)	0.790
Albumin, g/dL	141	2.7 (1.2–4.4)		335	3 (1.6–5.2)	<0.001
T. Bilirubin, mg/dL	141	1.9 (0.23–13.9)		335	1.8 (0.28–44.7)	0.723
AST, U/L	140	59 (9–822)		334	58 (16–7789)	0.718
ALT, U/L	140	35.5 (10–2088)		335	44 (11–3535)	0.001
ALP, U/L	140	121 (37–810)		334	117 (28–2327)	0.241
GGT, IU/L	140	67 (15–719)		334	73.5 (11–1396)	0.962
			HALP < 29 *n* (%)		HALP ≥ 29 *n* (%)	*p*
Gender	Female		25 (39.1)		39 (60.9)	0.075
	Male		116 (28.2)		296 (71.8)	
AFP ng/mL	≤200		113 (28.7)		281 (71.3)	0.371
	>200		26 (33.8)		51 (66.2)	
GGT, IU/L	≤104		88 (28.6)		220 (71.4)	0.531
	>104		52 (31.3)		114 (68.7)	
TTD, cm	≤8		91 (25.9)		260 (74.1)	0.003
	>8		50 (40)		75 (60)	
Differentiation	Well/Moderate		115 (28.6)		287 (71.4)	0.228
	Poor		26 (35.6)		47 (64.4)	
Venous invasion	None		70 (26.5)		194 (73.5)	0.098
	Micro+/Macro+		71 (33.5)		141 (66.5)	
CHILD class	A		30 (17.6)		140 (82.4)	<0.001
	B		69 (34.7)		130 (65.3)	
	C		42 (39.3)		65 (60.7)	
MELD-Na score	<15		74 (25)		222 (75)	0.005
	≥15		67 (37.2)		113 (62.8)	
Recurrence	None		96 (25)		288 (75)	<0.001
	Yes		45 (48.9)		47 (51.1)	
Milan Criteria	In		58 (23.9)		185 (76.1)	0.005
	Out		83 (35.6)		150 (64.4)	
Malatya Criteria	In		69 (22.9)		232 (77.1)	<0.001
	Out		72 (41.1)		103 (58.9)	
Expanded Malatya	In		78 (24)		247 (76)	<0.001
	Out		63 (41.7)		88 (58.3)	
GRWR, %	≥0.8		124 (30.1)		288 (69.9)	1.000
	<0.8		15 (29.4)		36 (70.6)	
Etiology	Viral hepatitis		96 (26.5)		266 (73.5)	0.018
	Cryptogenic		30 (42.3)		41 (57.7)	
	Ethanol		4 (44.4)		5 (55.6)	
	Budd-Chiari		5 (41.7)		7 (58.3)	
	Metabolik		0 (0)		8 (100)	
	Others		6 (42.9)		8 (57.1)	

Data are presented as median (minimum–maximum) for continuous variables and *n* (%) for categorical variables. AFP: alpha-fetoprotein; ALP: alkaline phosphatase; ALT: alanine aminotransferase; AST: aspartate aminotransferase; BSA: body surface area; BMI: body mass index; GGT: gamma-glutamyl transferase; GRWR: graft-to-recipient weight ratio; LTD: largest tumor diameter; NLR: neutrophil-to-lymphocyte ratio; PLR: platelet-to-lymphocyte ratio; TTD: total tumor diameter.

## Data Availability

The raw data used to support the findings of this study are available from the corresponding author upon request.

## Abbreviations

The following abbreviations are used in this manuscript:

AFP	Alpha-fetoprotein
ALP	Alkaline phosphatase
ALT	Alanine aminotransferase
AST	Aspartate aminotransferase
BSA	Body surface area
BMI	Body mass index
DFS	Disease-free survival
GGT	Gamma glutamyl transferase
GRWR	Graft-to-recipient weight ratio
HALP	Hemoglobin, Albumin, Lymphocyte, and Platelet score
HCC	Hepatocellular Carcinoma
LDLT	Living Donor Liver Transplantation
LT	Liver Transplantation
LTD	Largest tumor diameter
MELD	Model for End-stage Liver Disease
Macro+	Macrovascular invasion-positive
MASLD	Metabolic dysfunction-associated steatotic liver disease
Micro+	Microvascular invasion-positive
NLR	Neutrophil-to-lymphocyte ratio
OS	Overall Survival
PLR	Platelet-to-lymphocyte ratio
ROC	Receiver operating characteristic
TTD	Total tumor diameter

## Appendix A

**Table A1:** Demographic, Laboratory and Clinical data.

	*n*	Median (Min.–Max.)
Age at Tx date	476	56 (1–72)
BSA, mL/m^2^	476	1.91 (0.38–2.54)
BMI, kg/m^2^	476	26.01 (14.8–46.88)
AFP, ng/mL	471	12.3 (0.2–55,000)
LTD (cm)	476	3 (0–26)
Number of nodules	476	2 (1–36)
NLR	476	2.6 (0.15–35.3)
PLR	476	82.35 (2.61–1092.31)
Platelets, 10^3^/µL	476	97 (15–701)
INR	382	1.31 (0.82–4.1)
Creatinine, mg/dL	476	0.8 (0.37–13.8)
Albumin, g/dL	476	2.9 (1.2–5.2)
T. Bilirubin, mg/dL	476	1.84 (0.23–44.7)
AST, U/L	474	58.5 (9–7789)
ALT, U/L	475	43 (10–3535)
ALP, U/L	474	119 (28–2327)
GGT, IU/L	474	72.5 (11–1396)
		*n* (%)
Gender	Female	64 (13.4)
	Male	412 (86.6)
AFP, ng/mL	≤200	394 (83.7)
	>200	77 (16.3)
GGT, IU/L	≤104	308 (65)
	>104	166 (35)
TTD, cm	≤8	351 (73.7)
	>8	125 (26.3)
Differentiation	Well/Moderate	402 (84.6)
	Poor	73 (15.4)
Venous invasion	None	264 (55.5)
	Micro+/Macro+	212 (44.5)
CHILD class	A	170 (35.7)
	B	199 (41.8)
	C	107 (22.5)
MELD score	<15	296 (62.2)
	≥15	180 (37.8)
Recurrence	None	384 (80.7)
	Yes	92 (19.3)
Milan Criteria	In	243 (51.1)
	Out	233 (48.9)
Malatya Criteria	In	301 (63.2)
	Out	175 (36.8)
Expanded Malatya	In	325 (68.3)
	Out	151 (31.7)
GRWR, %	≥0.8	412 (89)
	<0.8	51 (11)
Etiology	Viral hepatitis	362 (76.1)
	Cryptogenic	71 (14.9)
	Ethanol	9 (1.9)
	Budd-Chiari	12 (2.5)
	Metabolik	8 (1.7)
	Others	14 (2.9)

Data are presented as median (minimum–maximum) for continuous variables and *n* (%) for categorical variables. AFP: alpha-fetoprotein; ALP: alkaline phosphatase; ALT: alanine aminotransferase; AST: aspartate aminotransferase; BSA: body surface area; BMI: body mass index; GGT: gamma-glutamyl transferase; GRWR: graft-to-recipient weight ratio; LTD: largest tumor diameter; NLR: neutrophil-to-lymphocyte ratio; PLR: platelet-to-lymphocyte ratio; TTD: total tumor diameter.

**Table A2:** Receiver operating characteristic (ROC) analysis of HALP score for recurrence.

Variable	AUC (95%, CI)	*p*-Value	Cut-off	Sensitivity (%)	Specifity (%)
HALP score	0.639 (0.594–0.683)	<0.001	≤28.9	50	75

AUC, area under the curve; CI, confidence interval. Optimal cut-off values were determined using the Youden index.

**Table A3:** Discriminative performance of selection criteria based on C-index and AUC.

Criterion	C-Index (95% CI)	AUC (95% CI)
HALP ≥ 29	0.565 (0.528–0.602)	0.581 (0.532–0.629)
Milan	0.621 (0.584–0.658)	0.628 (0.586–0.670)
Malatya	**0.649 (0.612–0.686)**	0.673 (0.629–0.716)
Expanded Malatya	0.648 (0.611–0.685)	**0.676 (0.630–0.721)**

AUC: area under the receiver operating characteristic curve; CI: confidence interval. The highest values for each metric are shown in bold.
